# Supplementary material for: Association between oral microbiome and seven types of cancers in East Asian population: a two-sample Mendelian randomization analysis
Source: Front Mol Biosci. 2023 Nov 21;10:1327893. doi: 10.3389/fmolb.2023.1327893 (PMC10702768; doi:10.3389/fmolb.2023.1327893)
Supplement: Supplementary file 1 [file DataSheet1.ZIP › Supplementary Materials/MR plots of saliva/saliva═╝/Prostate cancer/pheno.3154_to_prostate cancer_forest.pdf]

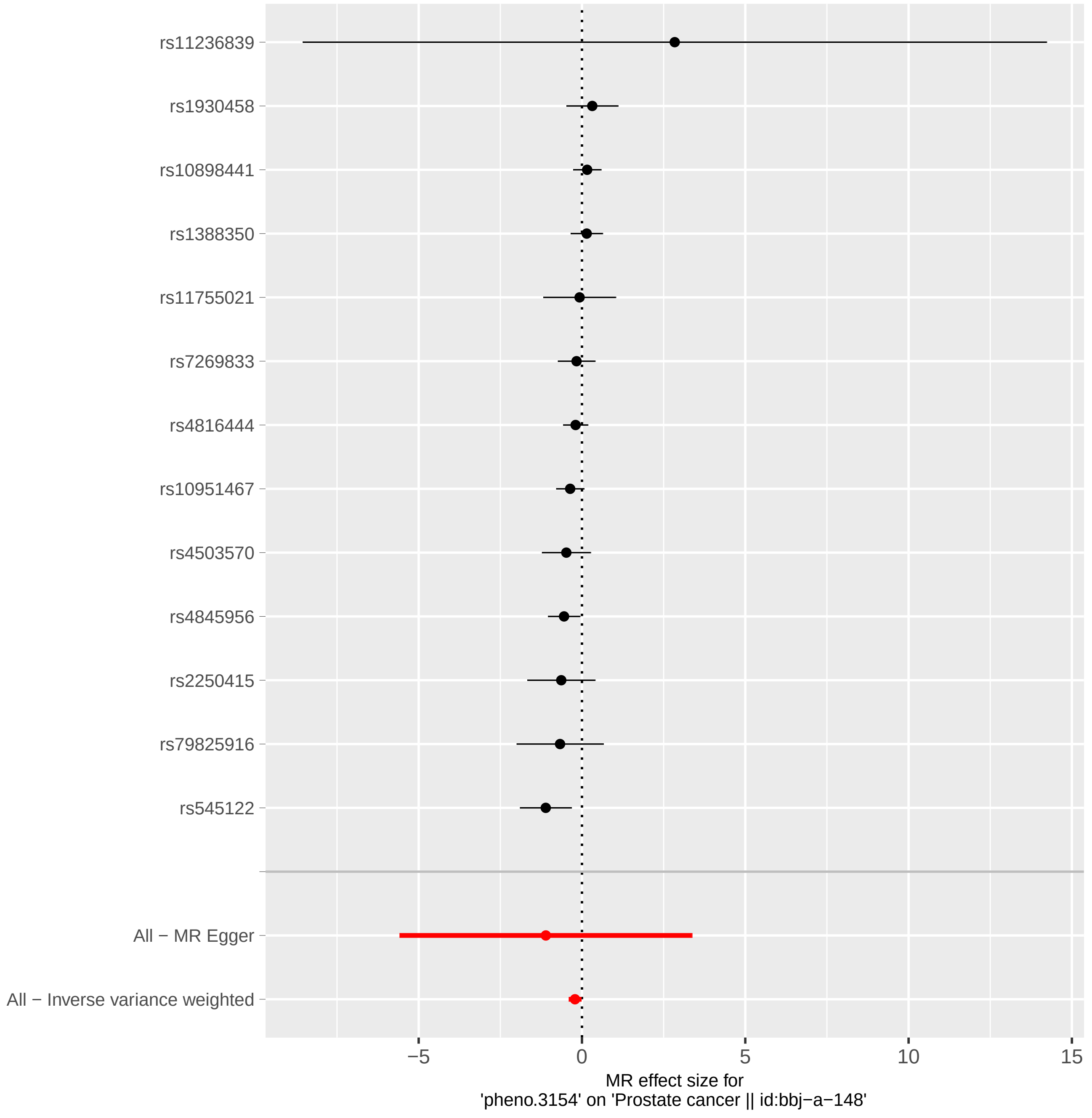
rs11236839
rs1930458
rs10898441
rs1388350
rs11755021
rs7269833
rs4816444
rs10951467
rs4503570
rs4845956
rs2250415
rs79825916
rs545122
All − MR Egger
All − Inverse variance weighted
−5
0
5
10
15
MR effect size for
'pheno.3154' on 'Prostate cancer || id:bbj−a−148'
